# Environmental factors affecting soil metals near outlet roads in Poznań, Poland: impact of grain size, soil depth, and wind dispersal

**DOI:** 10.1007/s10661-016-5284-5

**Published:** 2016-05-04

**Authors:** Jakub Ciazela, Marcin Siepak

**Affiliations:** Institut für Mineralogie, Leibniz Universität Hannover, Callinstr. 3, 30167 Hannover, Germany; Department of Hydrogeology and Water Protection, Institute of Geology, Adam Mickiewicz University, ul. Maków Polnych 16, 61-606 Poznań, Poland

**Keywords:** Soil pollution, Soil metal, Road transport, Grain size

## Abstract

We determined the Cd, Cr, Cu, Ni, Pb, and Zn concentrations in soil samples collected along the eight main outlet roads of Poznań. Samples were collected at distances of 1, 5, and 10 m from the roadway edges at depth intervals of 0–20 and 40–60 cm. The metal content was determined in seven grain size fractions. The highest metal concentrations were observed in the smallest fraction (<0.063 mm), which were up to four times higher than those in sand fractions. Soil Pb, Cu, and Zn (and to a lesser extent Ni, Cr, and Cd) all increased in relation to the geochemical background. At most sampling sites, metal concentrations decreased with increasing distance from roadway edges and increasing depth. In some locations, the accumulation of metals in soils appears to be strongly influenced by wind direction. Our survey findings should contribute in predicting the behavior of metals along outlet road, which is important by assessing sources for further migration of heavy metals into the groundwater, plants, and humans.

## Introduction

The main sources of soil pollution along roads include vehicle transport and the substances used in the winter maintenance of roads. In the first case, harmful organic and inorganic substances primarily originate from exhaust emissions, vehicle wear (e.g., tires, break shoes, and clutches), the corrosion of galvanized safety barriers, different types of leaks from vehicles, badly packed loads, and leaky tanks (Ahmed and Ishiga 2006; Johansson et al. 2009; Wei et al. 2009; Bojakowska et al. 2009). The main pollutants related to the road traffic include carbon, nitric and sulfur oxides, aromatic hydrocarbons, aldehydes, and metals. The latter include lead (the component of leaded petrol, which has commonly been used in Poland for several decades), zinc (coming from tire wear and used as an additive in motor oils), cadmium, and chromium. This list was augmented with platinum, palladium (used in the oxidation of carbon monoxide and hydrocarbons), and rhodium (used in the reduction of nitrogen oxides) in recent years (Merkisz and Kozak 2002; Bojakowska et al. 2009). The concentrations of these pollutants in the soil along roads depend on the traffic intensity, morphology of the area, local meteorological conditions, and soil properties (Warren and Birch 1987; Legret and Pagotto 2006). These pollutants can be transported by wind or the surface flow of precipitation water. They can also infiltrate into the groundwater. Metals can accumulate in the surface soil layer due to physical mechanisms (deposition and filtration of suspended solids) or as a result of physicochemical processes (adsorption and chelating; Legret and Pagotto 2006; Kabata-Pendias and Mukherjee 2007).

Urbanization and industrialization (both resulting in commensurate increases in road traffic intensity) rank among the primary contributors of anthropogenic metal pollution (e.g., Li et al. 2003; Duong and Lee 2009). Pb, Cu, and Zn concentrations in excess of the legal limit were noted in the park soils of Seville, the third biggest Spanish city (Madrid et al. 2002). The presence of these elements in city soils is believed to be mainly caused by road traffic, as is the case for Shanghai (Shi et al. 2008). In Kayseri—a large city in central Turkey with a population of almost one million—high Cd, Pb, and Zn concentrations have been observed in the soils located close to the main streets (Kartal et al. 2006). Wei and Young (2010) argue that Cd, Cu, Pb, and Zn concentrations in the soils of the biggest Chinese cities always exceed the values of geochemical backgrounds. However, this has not been confirmed for either Ni or Cr. Furthermore, both Ni and Cr show lower mobility (Duong and Lee 2009) and bioavailability than the other soil metals (Banerjee 2003).

Although the rate of industrial pollution is tending to decrease, road traffic continues to increase resulting in a commensurate increase in total pollution. In a study performed for Nanjing in western China, the Pb concentration in the humus layers of the soils along the sides of roads was augmented with respect to other city soils (parks, gardens, residential areas, and university campi). Furthermore, Pb concentrations correlated with Cr, Zn, and Cu concentrations (Lu et al. 2003). Such correlations were also noted in the atmosphere above the town of Urumqi in northwestern China. This was also interpreted as the effect of contamination by road traffic (Wei et al. 2009). The analysis of soil samples collected along Warsaw’s main outlet roads also showed that road traffic enhanced the concentrations of relevant metals in soils (Bojakowska et al. 2009).

Many investigations have been dedicated to the problem of soil metal concentrations in road dust (Ferreira-Baptista and Miguel 2005; Ahmed and Ishiga 2006; Duong and Lee 2009), showing the highest metal concentrations close to the margin of roads (up to 10–50 m). However, these investigations did not consider the effect of grain size on the accumulation of these metals in soils. The main objectives of this work are the following: (1) determination of the level of metal concentrations in soils near outlet roads in Poznań (Poland), (2) determination of the variability of concentrations depending on the distance from the road edge and the depth of sample collection, and (3) the analysis of the variability of metal concentrations in various granulometric fractions of the soil samples.

## Materials and methods

### Study area

**Fig. 1:**
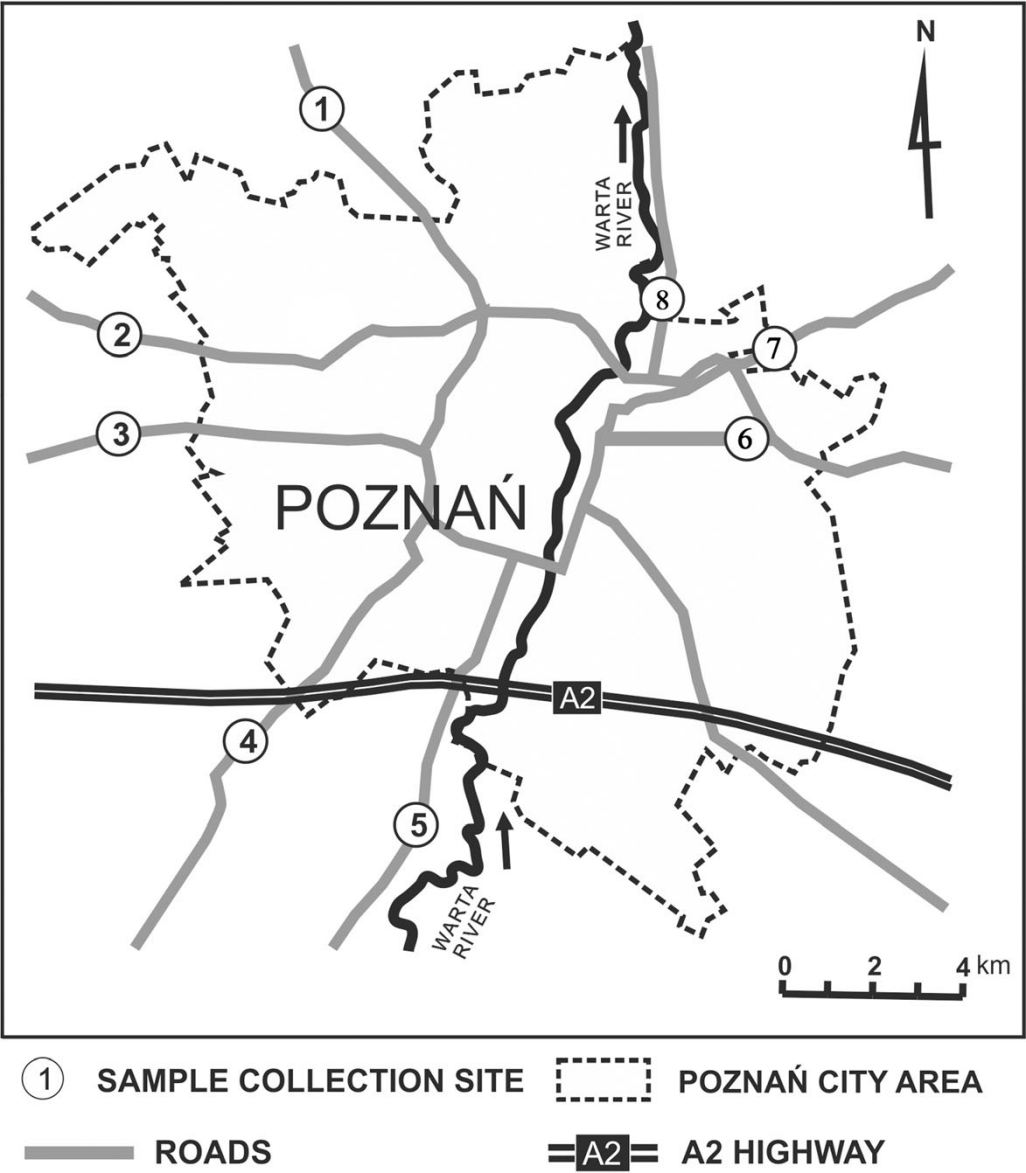
The map of the study area

Poznań, which covers an area of 262 km^2^, is the largest agglomeration in the western-central Poland (inset in Fig. 1) with a population of 550,000 inhabitants (Central Statistical Office 2014). The number is greatly increased by strong connection to 17 municipalities of the Poznań District in terms of economy and communication. It significantly contributes to the increasing intensity of road traffic in Poznań. The town has a temperate climate affected by a maritime airflow from the Atlantic Ocean and by a continental airflow from the east. The average annual temperature is 8.3 °C, with high seasonal variations (17.3 °C in the summer and −0.7 °C in the winter). This area is characterized by low precipitation, with an annual average of 529 mm (Woś 1994). The prevailing winds are westerly (inset in Fig. 4) but in the winter airflow can switch to a southeasterly direction (Jankowiak-Krysiak 2010).

### Sample collection and preparation

Ninety-six sandy soil samples were taken from eight locations along outlet roads in Poznań (Fig. 1 and Table 1) in the second half of March and the first half of April 2010. Twelve 500-g samples were collected in every location, at depths of 0–20 and 40–60 cm and distances of 1, 5, and 10 m from both road edges. Soils were taken with a 7-cm diameter Dutch auger and transported in polyethylene containers. After drying at room temperature for several days, the samples were divided into seven grain size fractions (Table 2) using a Retsch mechanical shaker (Germany). So, prepared 672 solid subsamples were extracted into solutions with aqua regia (HNO_3_/3HCl *v*/*v*; Merck, Germany) in a water bath for 1 h at 95 °C.

**Table 1:** Sampling locations and predominant soil types

No.	Street	Latitude (N)	Longitude (E)	Side of the road	Soil type
1	Obornicka	52° 28′ 51.04″	16° 51′ 50.93″	O	Bright sand
		52° 28′ 50.54″	16° 51′ 50.40″	I	Bright sand
2	J.H. Dąbrowskiego	52° 26′ 01.89″	16° 47′ 54.36″	O	Gravelly sand
		52° 26′ 00.60″	16° 47′ 53.82″	I	Bright sand
3	Bukowska	52° 24′ 51.90″	16° 46′ 31.04″	O	Gravelly sand
		52° 24′ 50.27″	16° 46′ 31.43″	I	Bright sand
4	Poznańska	52° 21′ 12.49″	16° 50′ 33.30″	O	Gravelly sand
		52° 21′ 11.42″	16° 50′ 34.87″	I	Bright sand
5	Armii Poznań	52° 19′ 14.75″	16° 53′ 06.17″	O	Dark gravelly sand
		52° 19′ 14.02″	16° 53′ 06.35″	I	Dark gravelly sand
6	Warszawska	52° 24′ 35.51″	16° 59′ 51.44″	O	Gravelly sand
		52° 24′ 38.46″	16° 59′ 52.14″	I	Bright sand
7	Gnieźnieńska	52° 25′ 41.59″	17° 00′ 14.19″	O	Sand
		52° 25′ 42.69″	17° 00′ 12.98″	I	Dark gravelly sand
8	Gdyńska	52° 26′ 24.78″	16° 58′ 57.06″	O	Gravelly sand
		52° 26′ 25.04″	16° 58′ 54.16″	I	Bright sand

*O* outward direction, *I* inward direction

**Table 2:** Particle size diameter (*d*_*p*_) ranges for each fraction size generated through dry sieving

Size fraction	Particle diameter (*d*_*p*_)
S1	*d*_*p*_ < 0.063 mm
S2	0.063 mm < *d*_*p*_ <0.1 mm
S3	0.1 mm < *d*_*p*_ <0.25 mm
S4	0.25 mm < *d*_*p*_ <0.5 mm
S5	0.5 mm < *d*_*p*_ <1 mm
S6	1 mm < *d*_*p*_ <2 mm
S7	2 mm < *d*_*p*_ <4 mm

### Chemical analysis

Soil metal and metalloid concentrations (Cd, Cr, Cu, Pb, Zn, Ni) were analyzed by atomic absorption spectrometry with flame atomization (F-AAS) using a Varian SpectrAA 280FS apparatus with an SPS3 autosampler. Elemental wave bands and lamp currents applied are listed in Table 3, together with the detection limits. The reagents used in the analyses were analytically pure, and the water was deionized to a resistivity of 18.2 MΩ cm in a Millipore Direct-Q® 3 Ultrapure Water System apparatus. To make calibration line, standard solutions were procured from Merck (Germany). Quality control of analytical measurements was performed using blank samples and the CRM027-050 certified material (Resource Technology Corporation, USA). The certified material was analyzed in six replicates. In Table 3, we provide medians of these analyses, together with standard deviations, as well as recovery rates computed by comparison with the certified values.

**Table 3:** Conditions and parameters of the analytical technique (F-AAS) used for determinations of Cd, Cr, Cu, Pb, Zn, and Ni, together with detection limits and results obtained for the certified reference material CRM027-050

Parameter		Cd	Cr	Cu	Pb	Zn	Ni
Wavelength	[nm]	228.8	357.9	213.9	217.0	213.9	232.0
Slit width	[nm]	0.5	0.2	1.0	1.0	1.0	0.2
Lamp current	[mA]	4.0	7.0	4.0	10.0	5.0	4.0
Oxid flow	[L/min]	12.7	11.5	12.7	12.7	12.7	12.7
Fuel flow	[L/min]	2.5	3.1	2.5	2.5	2.5	2.5
Sample flow rate	[mL/min]	5.0	5.0	5.0	5.0	5.0	5.0
Flame type		Air/acetylene					
Detection limit	[3σ; mg/kg]	0.01	0.02	0.01	0.03	0.02	0.03
Determination limit	[6σ; mg/kg]	0.03	0.06	0.03	0.09	0.06	0.09
CRM027-050 (*n* = 6)							
Certified value	[mg/kg]	12.0 ± 0.6	26.9 ± 1.7	9.87 ± 0.49	51.9 ± 2.5	51.3 ± 2.6	10.5 ± 0.7
Analyzed value	[mg/kg]	10.9 ± 0.4	24.3 ± 0.7	9.15 ± 0.49	48.5 ± 2.7	49.9 ± 0.5	9.92 ± 0.37
Recovery rate	[%]	91	90	93	93	96	94

Analyzed values are represented by medians with standard deviations after ±

**Fig. 2:**
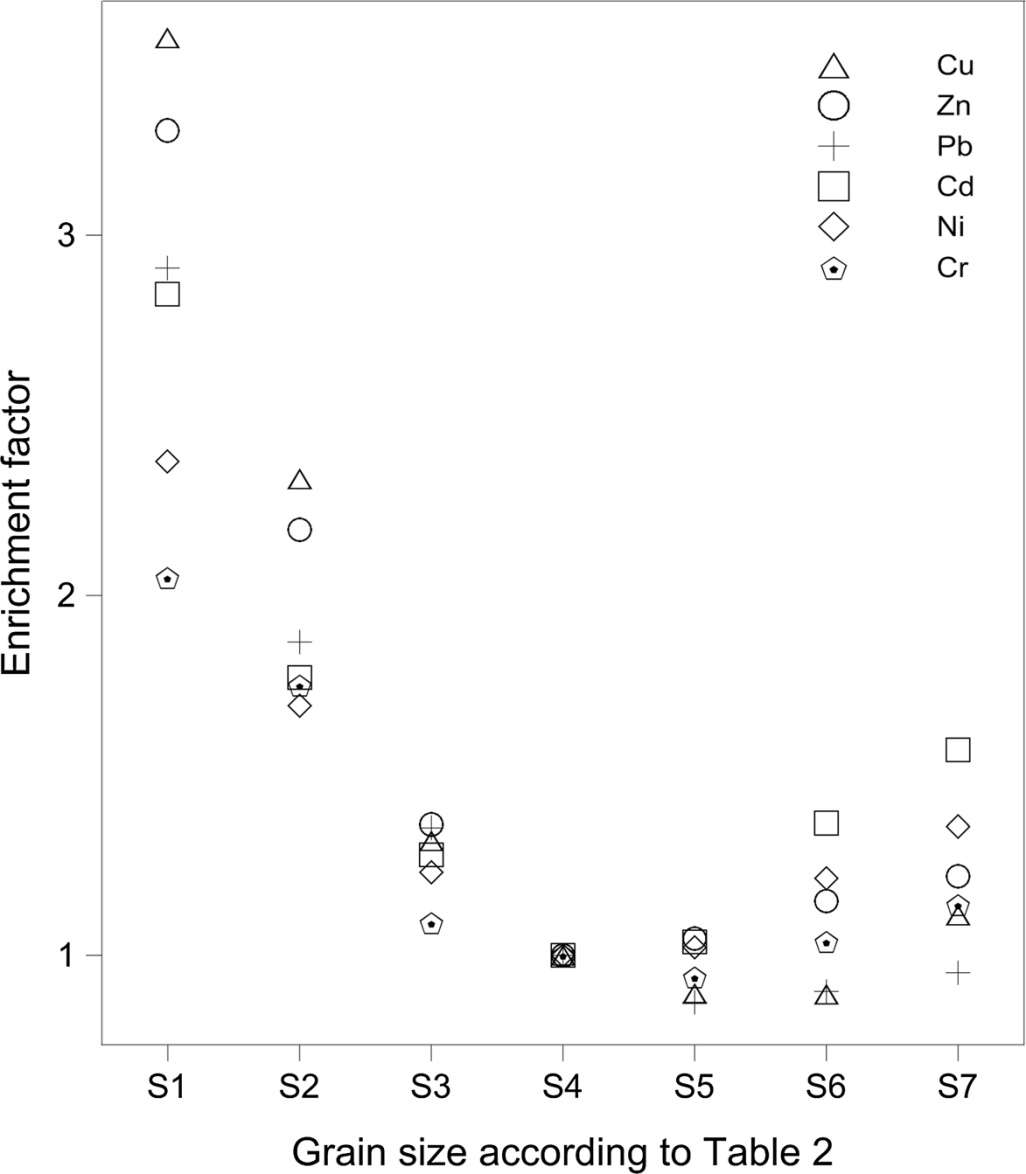
Metal concentration in dependence on grain size fraction. Symbols S1–S7 are described in Table 2. Enrichment factor is counted as a ratio of concentration in given fraction to fraction S4

The median concentrations of soil metals for the seven granulometric fractions (Table 2) have been calculated based on the samples collected over all the sampling sites, distances, and depths. In order to simplify further comparisons between the concentrations of various metals in various grain size fractions, the median values have been subsequently converted to an enrichment factor (EF), which is defined as follows. The EF of a given grain size (EF_i_) is calculated (Eq. 1) by dividing the median concentration of a given grain size (C_i_) through the median concentration of the grain size S4 (C_S4_; Table 2). Thus, the EF values for the grain size S4 always equal one. The grain size S4 has been chosen for this normalization as it is the most depleted in the majority of soil metals (Fig. 2).

1 $$ E{F}_i=\frac{C_i}{C_{S4}} $$

## Results and discussion

### Grain size factor

Grain size appeared to be the primary determinant of all the measured elements. The highest concentrations have always been observed in the smallest granulometric fraction S1. The lowest concentrations have been measured in intermediate fractions, S4 (Cd, Ni, Zn) and S5 (Cr, Cu, Pb; Fig. 2). The highest concentrations in the smallest fraction can be explained by their higher total absorption surface per mass, which decreases with increasing grain size. Consequently, the smallest metal concentrations should be expected in the largest grain size fractions. However, this is not the case for our set of samples as the soil metal concentrations in fractions S6 and S7 are slightly enhanced compared to S4 and S5 (Fig. 2). We think they are affected by the smallest grains cohesively aggregated on the surface of the largest grains, an effect which is difficult to eliminate during sieve analysis.

The highest median Cd concentration has been observed in the granulometric fraction S1 (1.10 mg/kg; EF = 2.84). The lowest concentrations have been found in the grain sizes S4 and S5 (0.41 and 0.43 mg/kg and EFs of 1.00 and 1.04, respectively). The largest grain size S7 displayed on average a lower Cd concentration (0.65 mg/kg; EF = 1.57). The Cd geochemical background for the Polish Lowland is 0.5 mg/kg (Pasieczna 2003).

The highest median of Cr concentrations (11.2 mg/kg; EF = 2.05) was observed also for the grain size S1, whereas the minimum value was found in the grain size S5 (5.2 mg/kg; EF = 0.94), being lower than the value for S4. The average concentration calculated for S7 was marginally higher (6.5 mg/kg; EF = 1.14). All three concentrations are higher than the average concentration measured in Poland, which is 4 mg/kg, and the geochemical background for the soils of the Polish Lowland (Pasieczna 2003), which is 3 mg/kg (Lis and Pasieczna 1995). The values, which are similar to ours, were noted along the roads of Warsaw (Bojakowska et al. 2009), whereas up to 30 times higher concentrations are exhibited in the soils along the roads of two Chinese cities, Beijing (57.5–65.5 mg/kg; Chen et al. 2010) and Xuzhou (67–162 mg/kg; Wang et al. 2007).

Relative differences in concentrations between various grain sizes are significantly higher for Cu in comparison to the differences for Cd and Cr. The smallest grain size fraction S1 shows an EF of 3.55 (20.3 mg/kg) with respect to S4, which is not even the most depleted in the case of Cu. In this case, the fraction S6 has the lowest concentration, exhibiting only 6.2 mg/kg Cu (EF = 0.89). All fractions, including the most depleted, are relatively enriched in comparison to the Cu geochemical background of Poland, being 5 mg/kg. Even so, these values are far lower than the concentrations shown by the soils along the roads of Beijing (on average 29.7 mg/kg; Chen et al. 2010) and Xuzhou (80 mg/kg; Wang et al. 2007), not mentioning the outlier value of 5727 mg/kg in the mine town of Kabwe in Zambia (Nakayama et al. 2011).

The Ni concentrations were on average highest in the smallest fraction S1 (13.3 mg/kg; EF = 2.37). This fraction covered a range between 3.59 and 40.4 mg/kg. The lowest concentrations have been observed in the fraction S4, having a median of 6.38 mg/kg. As in the case of the other elements, the Ni concentrations were slightly augmented in larger fractions with an enrichment factor of 1.36 in S7. The Ni geochemical background for Poland is 4 mg/kg (Lis and Pasieczna 1995) and thus less than any median concentration in any grain size interval. These values are far lower than the values determined for the soils along the roads of Beijing (26.7 mg/g; Chen et al. 2010). However, the geochemical background for Beijing is extremely high per se (26.8 mg/g; Chen et al. 2010), and thus the relative impact of traffic road pollutants on the average Ni concentration in the road soils is much higher in Poznań than in Beijing.

The lowest Pb concentrations have been observed in fraction S5, being on average 12.6 mg/kg (EF = 0.87). Similar to all the measured elements, Pb was the most enriched in fraction S1 with a concentration of 40.7 mg/kg and an enrichment factor of 2.91. The absolute enrichment factor, calculated as 2.91/0.87, was even higher (3.34), being one of the highest among the analyzed elements. The Pb concentrations in our samples were higher than the Pb Polish geochemical background for soils (16 mg/kg; Lis and Pasieczna 1995), although values were slightly lower than the values measured for Beijing (Chen et al. 2010; Table 4), Xuzhou (Wang et al. 2007), and Kabwe (Nakayama et al. 2011).

**Table 4:** Metal concentrations (μg/g) in soil samples along highway in various cities

City	Area	Parameter of sampling	Cu	Ni	Cd	Pb	Zn	Cr	Reference
Dhaka, Bangladesh	Commercial area (>1500 vehicles/h)	Dust from pavement edges	46^a^ ± 19^b^	26 ± 5	–	74 ± 36.4	154 ± 42.4	105 ± 16.9	Ahmed and Ishiga 2006
Delhi, India	Heavy traffic area (>5000 vehicles/h)	Eleven samples: at the intersection and 500 and 1000 m away in all the four directions	200	120	15	200	320	300	Banerjee 2003
Warsaw, Poland	Outlet roads	Depth of 0–20 cm, distances of 1.5 and 10 m	21	8	–	32	90	13	Bojakowska et al. 2009
Beijing, China	Ten main roads of the city	Distances of 1, 10, and 30 m	29.7 ± 5.7	26.7 ± 2.4	0.215 ± 0.070	35.4 ± 13.5	92.1 ± 18.7	61.9 ± 2.3	Chen et al. 2010
Kayseri, Turkey	Streets (not precisely defined)	Street dust swept directly from the road	99.1 ± 3.2	–	38.3 ± 1.1	1200 + _40	307 ± 4	–	Kartal et al. 2006
Nanjing, China	Roadside soil	Depths up to 1.2 m, grain sizes < 2 mm	117.3 ± 83.4	–	–	151.4 ± 68.2	280.3 ± 194.3	88.6 ± 20.3	Lu et al. 2003

^a^Arithmetic mean

^b^Standard deviation

The concentrations of Zn were highest in fraction S1 (73 mg/kg; EF = 3.29). The lowest Zn concentrations have been measured in fraction S5, being 24.8 mg/kg. As in the case of the other elements, the median concentration in the largest fraction S7 was slightly higher (31.4 mg/kg; EF = 1.22; Fig. 2). For comparison, the geochemical background for the Polish Lowland is 25 mg/kg (Lis and Pasieczna 1995) and the geometrical mean for Poland is 40 mg/kg (Pasieczna 2003). Thus, the concentrations of Zn in Poznań’s roadside soils are moderately augmented in relation to the other soils. However, much more enhanced Zn concentrations have been observed in the two Chinese cities, Beijing (from 66 to 138 mg/kg; Chen et al. 2010) and Xuzhou (from 83 to 380 mg/kg; Wang et al. 2007).

### Soil depth factor

Soil metal concentrations were highest in the top (0–20 cm) portion of soils (Table 5). The relative difference between the top and the deeper (40–60 cm) portion of soil was typically most pronounced at the distance of 1 m, at the lowest at the distance of 5 m. For example, the Pb concentration in the interval of 0–20 cm for the distance of 1 m (108.0 mg/kg) exceeded more than twice the Pb concentration in the interval of 40–60 cm for the same distance (50.1 mg/kg). Such a strong difference has not been observed for the distances of 5 m, where it was only 31 %. The excess of Cd in the surface value with respect to the median calculated for the samples collected at depth was 84, 41, and 77 % for the distances of 1, 5, and 10 m, respectively. Similarly for Zn, whereas the differences between the two depth intervals were only 33 % at the distance of 5 m, the differences were very high at the distances of 1 and 10 m, exceeding 100 % at the latter distance. A similar but “flattened” pattern was displayed by Cr, with the values of 59, 28, and 39 %. Notably, the median Cu concentrations decreased by a similar degree (39, 43, and 52 %, respectively) at all the three distances. A similar but reversed systematics has been determined for Ni. This element was enriched in the surface interval by 40, 41, and 23 % for the distances of 1, 5, and 10 m, respectively.

**Table 5:** Metal concentrations (mg/kg) measured in fraction S1 depending on depth (rows) and distance from the road (columns)

Parameter	Element	Cu			Ni			Cd			Pb			Zn			Cr		
	Depth (cm)/distance (m)	1	5	10	1	5	10	1	5	10	1	5	10	1	5	10	1	5	10
Median	0–20	63.9	35.4	28.1	27.3	20.1	11.7	2.19	1.45	1.07	108.0	49.1	39.9	156.2	106.2	82.4	21.5	13.4	9.86
	40–60	46.1	24.7	18.4	19.1	14.3	9.50	1.19	1.03	0.60	50.1	37.4	22.7	90.2	79.9	40.9	13.5	10.5	7.08
Standard deviation	0–20	34.1	25.7	19.6	9.62	8.34	5.83	0.75	0.57	0.37	44.5	25.3	20.8	136.2	83.8	46.8	20.1	5.49	3.89
	40–60	33.0	18.4	14.6	9.33	7.85	5.09	0.64	0.33	0.50	35.4	17.0	12.9	84.1	53.0	33.6	7.63	4.31	4.22
Minimum	0–20	5.57	1.51	0.84	9.26	6.42	5.17	1.05	0.51	0.54	24.7	22.7	12.7	70.6	16.0	8.23	8.10	6.67	3.96
	40–60	1.94	1.43	1.35	7.05	5.92	3.59	0.50	0.44	0.35	23.3	14.7	7.34	28.6	11.9	7.20	7.40	5.35	2.72
Maximum	0–20	117.9	93.5	74.0	40.4	31.2	22.9	3.64	2.37	1.91	182.1	110.2	83.8	505.1	290.8	193.2	91.6	24.3	19.7
	40–60	99.5	70.6	53.7	36.8	28.9	20.0	2.49	1.64	2.33	114.7	72.3	62.8	302.2	192.7	147.0	31.2	20.3	19.9

**Fig. 3:**
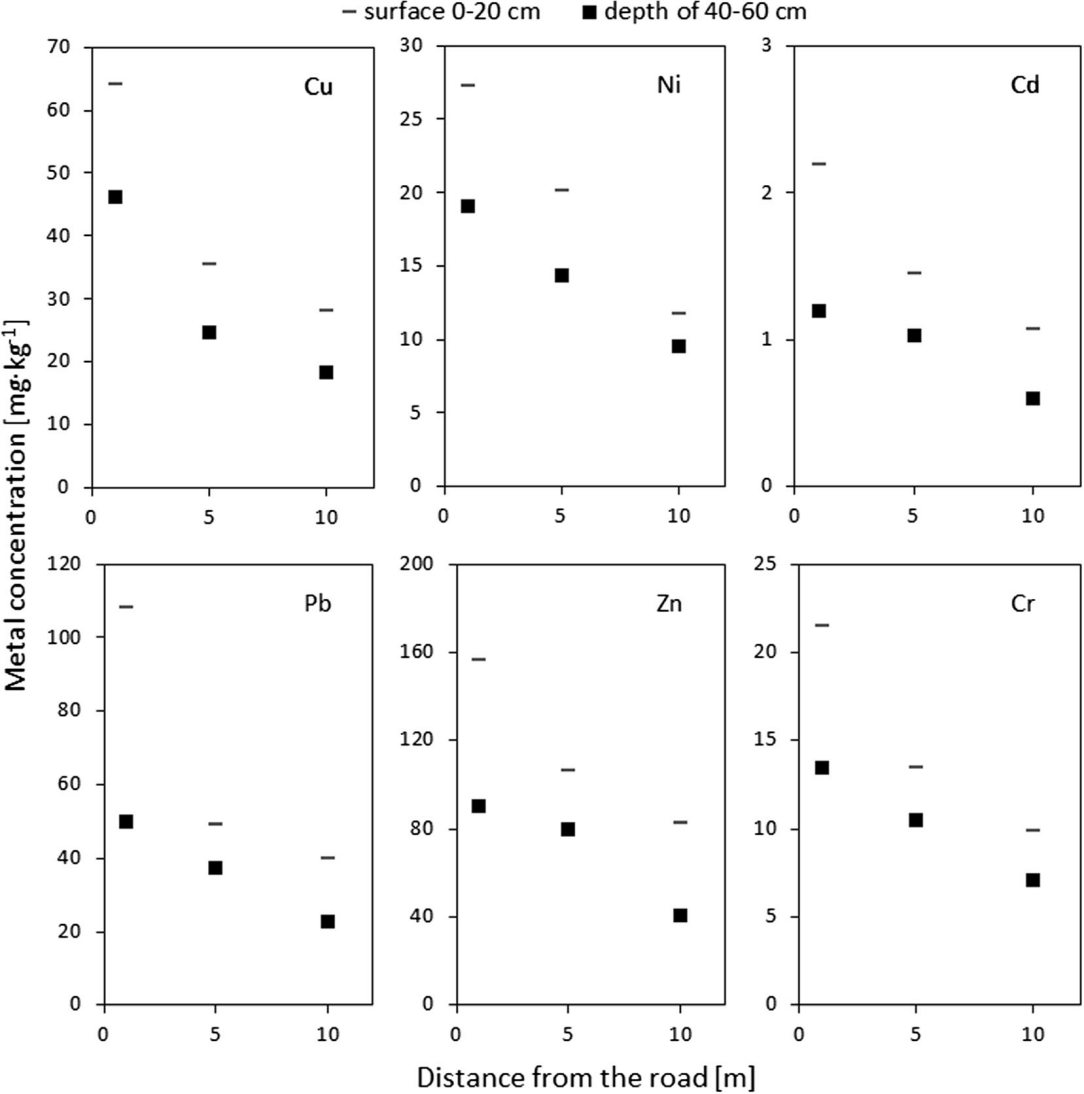
Metal concentration depending on depth (*horizontal lines* and *squares*) and distance from the road (*horizontal axis*)

Considering the abovementioned values and the trends presented in Fig. 3, the largest decrease of soil metal concentrations is likely found in the road-proximal zone (<5 m). Thus, an exponential growth of soil metal concentrations in surface layers can be expected approaching the road margin. The reverse is observed for the material collected at depth. Approaching the road margin, a logarithmic growth is primarily observed, with soil metal concentrations growing relatively slowly in the proximity of the road. Inflection points of both curves are located at the distance of ~5 m from the margin of the road, and thus the difference in concentrations displayed by the two depth intervals is relatively low at this distance.

A similar negative correlation between soil metal content and depth was also observed in two other studies, performed in Warsaw (Bojakowska et al. 2009) and Istanbul (Guney et al. 2010). Guney and coworkers provided the average concentrations for surface soil samples (0–2 cm) and 19–21-cm depth soil samples collected along highways, within 1-m distance from the edge of the pavement. The average concentrations of Pb, Zn, and Cu in the surface layer were 191, 255, and 69 mg/kg, respectively. At 20 cm below the surface, the average concentrations were 81, 211, and 47 mg/kg, respectively. Then, the decrease of the Pb concentration with depth is 57 %/19 cm. The same parameter is 17 %/19 cm in the case of Zn and 32 %/19 cm in the case of Cu. For comparison, the analogous gradients calculated for our soil samples are 54 %/40 cm for Pb, 42 %/40 cm for Zn, and 28 %/40 cm for Cu. These values are similar to each other, but they are produced on a steeper gradient in the case of Istanbul. One reason for that can be the fact that a decrease in concentration caused by road traffic should be exponential and most rapid in the shallowest layer of a ground. Thus, concentration differences measured in relation to depth differences should indeed be higher for the uppermost layer of the soil (1- to 20-cm depth as in the case of Istanbul) than in a lower layer of the soil (10- to 50-cm depth as in our study). The gradients for Pb are higher than for the other elements in both studies. This fact is an indication that road traffic has generally a relatively higher impact on Pb concentrations than Zn and Cu concentrations in the soils adjacent to roads. This is also the case for Warsaw, where gradients for Pb are 50 %/40 cm, being slightly higher than those for Zn (47 %/40 cm) and Cu (46 %/40 cm). The values for Zn are relatively low in Istanbul with respect to the values for the other elements and the values from our study and Warsaw’s study. This likely accounts for relatively elevated geochemical background (~90 mg/kg; Guney et al. 2010) in Istanbul. After subtracting the background value from the absolute values at both depths, the Zn gradient would be 27 %/19 cm, fitting better in the presented dataset.

### Distance factor

Decreasing concentrations of all the measured metals with increasing distance to the road edge have been observed. In all cases except for Ni, the drop in concentration is more pronounced at the 1–5-m interval than 5–10-m interval for the surface layer. The opposite is true for the deeper layer, where only Cu exhibits a similar systematics (Fig. 3).

The median Cd concentration in the surface layer 1 m from the road edge is 2.19 mg/kg, whereas it is only 1.07 mg/kg at the distance of 10 m. This means a 50 % decrease. A similar decrease was noted at the deeper layer. Whereas the Cd concentration at the distance of 1 m is 1.19 mg/kg, it is only 0.60 mg/kg at the distance of 10 m. The median Cr concentration at the surface layer is 21.47 mg/kg at the distance of 1 m and 9.86 mg/kg at the distance of 10 m, which means an even higher decrease (54 %) than in the case of Cd. One of the most pronounced difference has been found in Cu concentration. The Cu concentration at the distance of 10 m is 60 % lower than at the distance of 1 m at the surface layer. A decrease of 56 % has been noted at the deeper depth interval.

The median Ni concentration in the surface layer was 27.3 mg/kg next to a road edge and only 11.7 mg/kg at the distance of 10 m, which means a decrease of 55 %. Even a higher decrease has been observed for Pb, which has been found abundant at the surface layer near to the road edge (108.0 mg/kg) and in a level of 39.9 mg/kg at the distance of 10 m. A smaller decrease has been identified in the deeper layer, where the values were 50.1 and 22.7 mg/kg, respectively. These numbers mean a decrease of 65 % in the surface layer and 55 % in the deeper layer. Interestingly, the opposite was found for Zn. While the Zn concentration decreases by 55 % between 1- and 10-m distance at the deeper layer, as in the case of Pb, it decreases only by 45 % in the surface layer.

The general decrease of soil metal concentrations with increasing distance from the road edge is consistent with the results of studies performed for other heavy traffic areas. They refer to urbanized (Bojakowska et al. 2009; Chen et al. 2010) as well as non-urbanized areas (Earon et al. 2012). For example, in Warsaw (Bojakowska et al. 2009), the Pb concentrations are higher at 1 m than 10 m from the margin of road in 11 of 15 sampling sites. This is also the case for Zn in 10 of 15 sampling sites and for Ni in 9 of 15 sampling sites. In Beijing, this effect was observed in 9 of 10 presented sampling sites for Pb, in all the sites for Zn, but only in one for Ni (Chen et al. 2010). The latter is surprising as we observe an average 60 %/9-m decrease of Ni concentration in our sample set. One explanation for that is significantly higher geochemical background in Beijing in comparison to the one in Poznań (see “Grain size factor” section).

### Wind factor

**Fig. 4:**
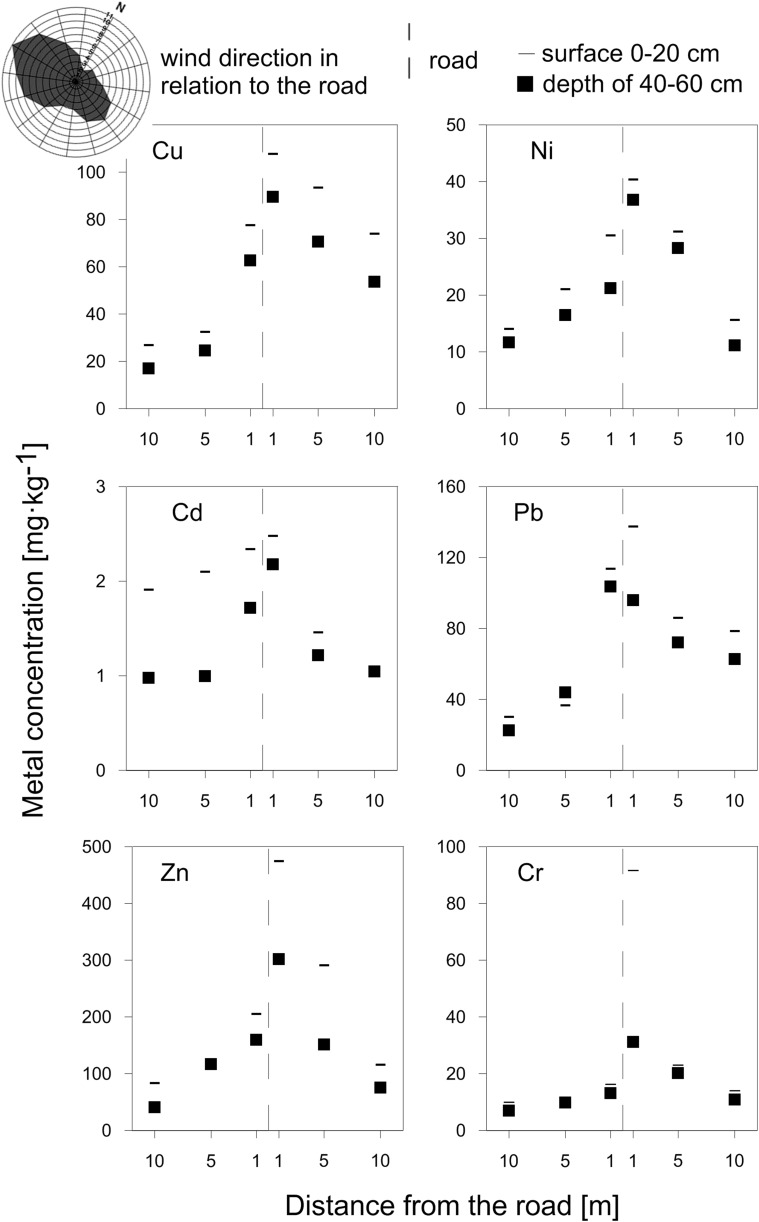
Distribution of metal concentration at site 5 (Armii Krajowej). The orientation of the road is close to N-S. The higher concentrations of most soil metals are observed on the eastern side of the road. The wind rose in the *top left corner* shows the domination of westerly winds, which probably affects the distribution of the road pollutants

The prevailing winds in Poznań are westerly (inset in Fig. 4). Thus, we should expect higher soil metal concentrations on the eastern sides of the north-south (N-S) oriented roads, if this distribution is affected by winds. The following roads are oriented in an N-S direction: Obornicka (site 1), Wrocławska (site 4), and Armii Poznań (site 5; Fig. 1). We have determined the soil metal concentrations in the fraction of <0.063 mm at the distance of 1, 5, and 10 m at the eastern and western sides of these roads. The concentration distributions for all three sites show a similar systematics (presented in Fig. 4 for site 5). The higher concentration of most soil metals can be observed at the eastern side of the road (Table 6). This effect is the most pronounced at the lowest distance of 1 m (Fig. 4).

**Table 6:** Metal concentration (mg/kg) at the surface layer of the soils and a distance of 1 m from the road on the western (W) and eastern (E) sides of the roads

Street (site number)	Element	W	E
Obornicka (1)	Cu	0.9	**2.0**
	Cd	1.0	**2.1**
	Ni	5.6	**11.4**
	Pb	22.6	**111.1**
	Zn	36.0	**124.2**
	Cr	12.5	**23.2**
Poznańska (4)	Cu	24.2	**28.6**
	Cd	**2.0**	1.1
	Ni	9.3	**10.4**
	Pb	24.7	**43.9**
	Zn	70.6	**86.6**
	Cr	8.1	**11.3**
Armii Poznań (5)	Cu	77.6	**107.8**
	Cd	2.3	**2.5**
	Ni	30.5	**40.4**
	Pb	113.6	**137.5**
	Zn	205.5	**474.2**
	Cr	16.2	**91.6**

Numbers in brackets are according to the map in Fig. 1. Higher values (bolded) are generally on the eastern side

The effect of wind direction on soil metal distribution in road side soils has also been investigated by Chen et al. (2010) in Beijing. The dominance of one wind direction (northwest) in Beijing is not that clear as in Poznań making such a research more difficult. Even so, Chen and coworkers also provided evidences for increased concentrations of Pb, Zn, Cu, and Cd in the downside directions at two sampling sites along the NE-SW oriented roads. Moreover, this effect was visible even at the distance of 30 m in one of these sites.

## Conclusions

1. Road transport significantly increases soil metal concentrations in soils adjacent to heavy traffic roads. Pb, Cu, and Zn concentrations are highly enriched by this process, whereas Ni and Cr are moderately enriched. Cd concentrations are affected by road transport only in the proximity of the road (~1 m). In Poznań, a city with a low degree of industrialization, the relative influence of road transport on the heavy metal concentrations in roadside soil is more pronounced than in cities with higher degree of industrialization like Beijing.
2. Soil metals are especially enriched at a low distance (<5 m) from the road at the surface layer of soils. However, winds can widen this zone and additionally increase soil metal concentrations.
3. The soil metal concentrations rapidly decrease with depth. Especially the Pb concentration can drop by more than twice within 50 cm.
4. The concentrations of all analyzed metals strongly depend on the granulometric structure of soils. Metals are especially enriched in the smallest granulometric fractions, where the aggregated surface of grains provides more positions to occupy. This effect is most pronounced for Zn and Cu with up to four times higher concentrations in silts than in sands.
5. These findings should contribute in assessing sources for further migration of heavy metals into the groundwater, crops, garden vegetables, and finally humans. Moreover, zoning plans should consider these results when planning new agricultural lands, single family houses, and green areas close to major roadways.
